# Study of serious adverse drug reactions using FDA-approved drug labeling and MedDRA

**DOI:** 10.1186/s12859-019-2628-5

**Published:** 2019-03-14

**Authors:** Leihong Wu, Taylor Ingle, Zhichao Liu, Anna Zhao-Wong, Stephen Harris, Shraddha Thakkar, Guangxu Zhou, Junshuang Yang, Joshua Xu, Darshan Mehta, Weigong Ge, Weida Tong, Hong Fang

**Affiliations:** 1 National Center for Toxicological Research, U.S. Food and Drug Administration, Jefferson, AR 72079 USA; 2 MedDRA Maintenance and Support Services Organization, 7575 Colshire Dr., McLean, VA 22102 USA

**Keywords:** Adverse drug reactions, Data mining, MedDRA, Drug labeling, Boxed Warning, Structured product labeling, Standard terminology, Drug safety

## Abstract

**Background:**

Adverse Drug Reactions (ADRs) are of great public health concern. FDA-approved drug labeling summarizes ADRs of a drug product mainly in three sections, i.e., Boxed Warning (BW), Warnings and Precautions (WP), and Adverse Reactions (AR), where the severity of ADRs are intended to decrease in the order of BW > WP > AR. Several reported studies have extracted ADRs from labeling documents, but most, if not all, did not discriminate the severity of the ADRs by the different labeling sections. Such a practice could overstate or underestimate the impact of certain ADRs to the public health. In this study, we applied the Medical Dictionary for Regulatory Activities (MedDRA) to drug labeling and systematically analyzed and compared the ADRs from the three labeling sections with a specific emphasis on analyzing serious ADRs presented in BW, which is of most drug safety concern.

**Results:**

This study investigated New Drug Application (NDA) labeling documents for 1164 single-ingredient drugs using Oracle Text search to extract MedDRA terms. We found that only a small portion of MedDRA Preferred Terms (PTs), 3819 out of 21,920 or 17.42%, were observed in a whole set of documents. In detail, 466/3819 (12.0%) PTs were in BW, 2023/3819 (53.0%) were in WP, and 2961/3819 (77.5%) were in AR sections. We also found a higher overlap of top 20 occurring BW PTs with WP sections compared to AR sections. Within the MedDRA System Organ Class levels, serious ADRs (sADRs) from BW were prevalent in Nervous System disorders and Vascular disorders. A Hierarchical Cluster Analysis (HCA) revealed that drugs within the same therapeutic category shared the same ADR patterns in BW (e.g., nervous system drug class is highly associated with drug abuse terms such as *dependence*, *substance abuse*, and *respiratory depression*).

**Conclusions:**

This study demonstrated that combining MedDRA standard terminologies with data mining techniques facilitated computer-aided ADR analysis of drug labeling. We also highlighted the importance of labeling sections that differ in seriousness and application in drug safety. Using sADRs primarily related to BW sections, we illustrated a prototype approach for computer-aided ADR monitoring and studies which can be applied to other public health documents.

**Electronic supplementary material:**

The online version of this article (10.1186/s12859-019-2628-5) contains supplementary material, which is available to authorized users.

## Background

Adverse Drug Reactions (ADRs) are harmful events related to the use of a drug product. A serious Adverse Drug Reaction (sADR) is defined as any event or reaction that results in death, a life threatening adverse event, inpatient hospitalization or prolongation of existing hospitalization, a persistent or significant incapacity or substantial disruption of the ability to conduct normal life functions, or a congenital anomaly or birth defect [1, 2]. In the U.S., sADRs contribute to over 100,000 deaths per year and have been one of the leading causes of mortality over the past several decades, and thus impose a significant public health concern [1, 3–7]. sADRs such as liver failure and fatal arrhythmia, can lead to a drug being withdrawn from the market when the risks outweigh the benefits [8–11].

FDA-approved drug labeling is defined by the Code of Federal Regulations (21CFR201.57) [12] and contains 17 distinct sections. Each section provides specific information such as drug safety (e.g., Drug Interactions and Contraindications), efficacy (e.g., Indications & Usage and Dosage & Administration), patient information (e.g., Patient Counseling Information), target populations (e.g., Use in Specific Populations), and clinical and nonclinical data (e.g., Clinical Pharmacology and Nonclinical Toxicology) [13]. To promote the safe use of drug products and protect public health, ADR information is collected from clinical trials and post-marketing surveillance data and summarized in FDA-approved drug labeling [14]. Boxed Warning (BW), Warnings and Precautions (WP), and Adverse Reactions (AR) are three sections that focus on ADRs.

Even though these three sections involve ADRs, each has a different level of severity and coverage. BW describes “*serious warnings, particularly those that lead to death or serious injury,*” while WP describes “*clinically significant adverse reactions*,” and AR describes “*overall adverse reaction profile of the drug*” [12]. Consequently, ADRs mentioned in BW are the most serious, whereas those in either WP or AR contain serious and less-serious ADRs. While each of these three sections do contain pertinent information related to adverse reactions that is valuable and critical for health professionals to promote the safe use of the drug product. Overall, if these three ADR related sections are treated equally could lead to an inadequate assessment of the severity degree of ADRs, and could lead to misinterpretation or unintended harmful events. Therefore, it is important to consider the different levels of severity associated with labeling sections when studying ADRs.

The Medical Dictionary for Regulatory Activities (MedDRA) [15–18] is the standard medical terminology developed by the International Council for Harmonization (ICH) of Technical Requirements for Pharmaceuticals for Human Use, and is used worldwide to facilitate the sharing of regulatory information for medical products. MedDRA is mandated in Europe and Japan for safety reports [19], and has been used for coding adverse events in the FDA’s Adverse Event Reporting System (FAERS) [20]. MedDRA is widely applied in analyzing adverse event report data [21–24] and in mining public health data (e.g., Medline, WebMD, and Web of Science databases) for potential safety concerns [25–28]. One of the key features of MedDRA is its five-level hierarchical structure. The basic Low Level Terms (LLTs) are the most granular terms and can be used to encode adverse events (AEs) or ADRs. LLTs often include common and well known terms that patients, those reporting ADRs, and some healthcare providers frequently use. Synonymous and quasi-synonymous LLTs are grouped under a Preferred Term (PT), which many health care providers and researchers are prone to use. Through the hierarchy, clinically relevant PTs are grouped under High Level Terms (HLT), and relevant HLTs are grouped under High Level Group Terms (HLGT) in System Organ Classes (SOC). This network of linked terms provides a method to standardize the language used and allows for accurate analysis of reported ADRs.

Studies have successfully implemented the use of MedDRA terminology to code and investigate ADRs in a variety of documents. For example, a study conducted by *Thiessard* et al. applied MedDRA terminology to study over 190,000 ADR reports in the French spontaneous reporting system between years 1986–2001 [21] and discovered that ADRs related to skin and subcutaneous tissue disorders and nervous system disorders were the most frequently reported*. de Langen* et al. used MedDRA to code and compare ADRs self-reported by patients and those reported by healthcare professionals, to evaluate the intrinsic value of patient self-reporting [22], and found differences in the categories of the seriousness (e.g., life-threatening and death related ADRs).

MedDRA has also been used to analyze ADRs in FDA drug labeling [29, 30]. For example, the Side Effect Resource Database (SIDER) applied MedDRA terminology to extract ADR information from drug labeling [30–32]. In our previous research, we have applied MedDRA to drug labeling to assess the utility of ADRs in drug repurposing [33]. However, most research on drug labeling, if not all, does not discriminate the severity of an ADR according to different labeling sections (e.g., BW, WP, and AR). Therefore, they might not provide an adequate assessment of drug toxicity and severity, potentially undermining the utility of drug labeling.

To demonstrate the utility of FDA-approved drug labeling for the study of ADRs, we compared the results from the three sections with a specific focus on sADRs presented in BW. Our results demonstrate that this computer-aided ADR analysis of combining standardized terminology of MedDRA with data mining techniques allowed us to characterize the frequency, severity, and pattern of ADRs in drug labeling documents. This approach provides a prototype for the study of ADRs in other public health documents.

## Results

### ADR analysis based on different drug labeling sections

Of the 1164 New Drug Application (NDA) labeling documents analyzed, 31.5% contained Boxed Warnings (BW, 367), while over 98% had Warnings and Precautions (WP, 1148) and Adverse Reactions (AR, 1152) sections. We used Oracle Text search to extract MedDRA Low Level Terms (LLTs) from the documents, which were further mapped to their corresponding Preferred Terms (PTs) based on MedDRA hierarchy. A total of 3819 out of 21,920 (17.42%) MedDRA PTs were identified within the whole labeling body of the 1164 documents. PT analysis by section revealed that 460/3819 PTs (12%) occurred in BW sections, 2013/3819 occurred in WP (53.0%) and 2961/3819 occurred in AR (77.5%) (Table 1). The entire corpus for Boxed Warning sections among these drugs is provided in Additional file 1.

**Table 1:** Occurrence of MedDRA terms in three ADR related labeling sections

ADR Section Name	# Drugs	# Low Level Terms*	# Preferred Terms*
Boxed Warning (BW)	367	593 (*8.1%*)	460 (*12.0%*)
Warnings and Precautions (WP)	1148	3206 (*44.0%*)	2023 (53.0*%*)
Adverse Reactions (AR)	1152	5300 (*72.7%*)	2961 (*77.5%*)
Whole Labeling Document	1164	7287 (*100.00%*)	3819 (*100.00%*)

Total number of LLTs is 75,818 and PTs is 21920 in MedDRA, version 19.0

*Only ADR related 22 disorder SOCs was investigated

**Fig. 1:**
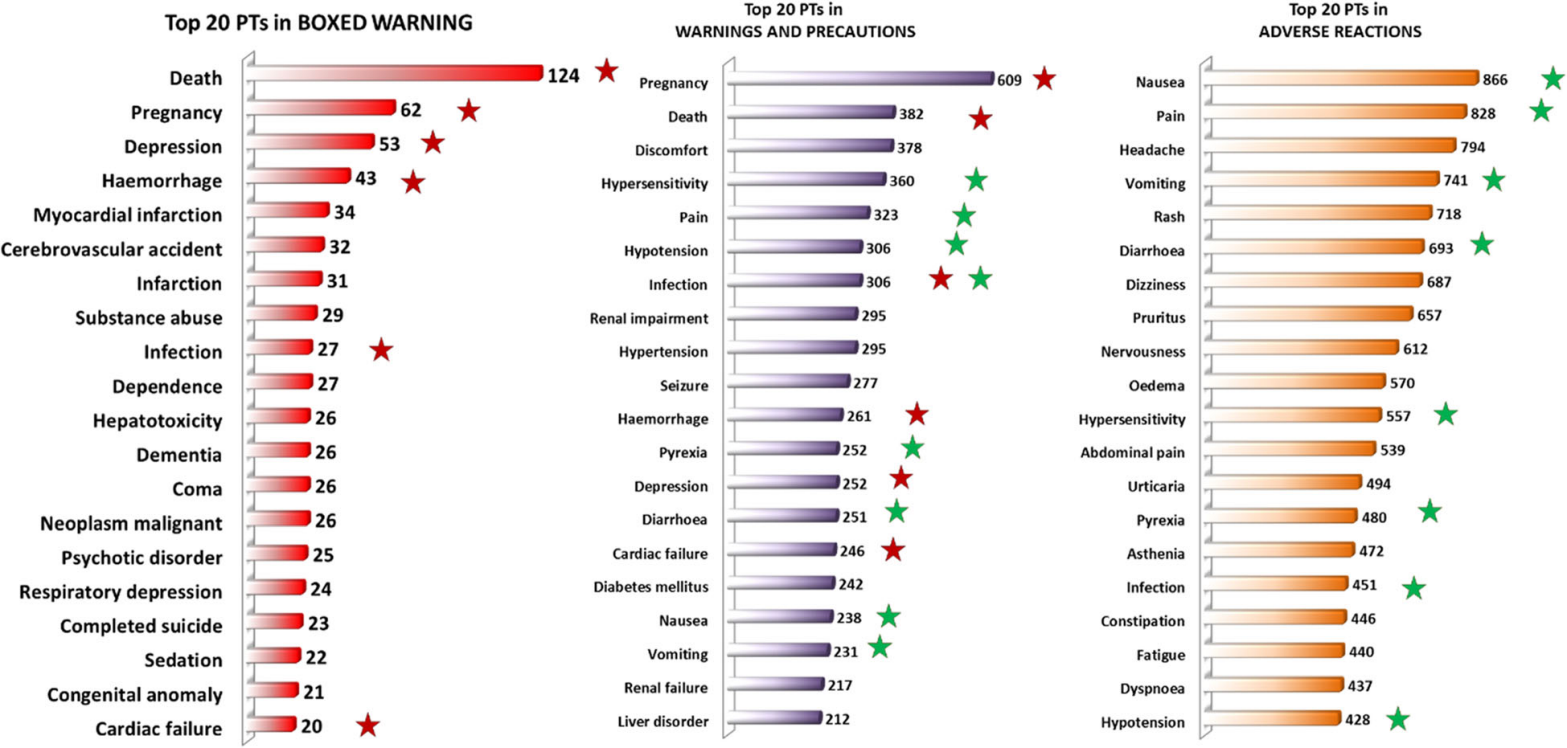
Top 20 PTs observed in BOXED WARNING (BW), WARNINGS AND PRECAUTIONS (WP), and ADVERSE REACTIONS (AR) sections among labeling of 1164 drugs. Overlapped PTs between BW and WP/AR sections are highlighted as follows: 6 PTs overlapped between BW and WP (red stars), 8 PTs overlapped between WP and AR (green stars), only 1 PT (*infection*) overlapped between BW, WP and AR

To investigate a more detailed PT distribution across labeling sections, we compared the top 20 most observed PTs of the BW, WP, and AR sections each. As shown in Fig. 1, the most frequently present PT in BW was *Death* (observed 124 times). Upon comparing BW and WP sections, we identified six overlapping PTs (*Death*, *Pregnancy*, *Depression*, *Hemorrhage*, *Cardiac failure,* and *Infection*; red stars in Fig. 1) among the top 20s. In contrast, we only observed one overlapping PT (*Infection*) when we compared BW and AR. Of note, eight PTs (green stars in Fig. 1) overlapped between WP and AR; most of these ADRs are not sADRs and are associated with symptoms rather than actual severe adverse events or diseases. Thus, they are high in frequency but relatively less serious ADRs compared to sADRs. This supports the claim that BW, WP, and AR sections have different focuses, with BW focusing on sADRs.

These results further support our theory that by simply treating these three ADR sections equally could lead to the misinterpretation and potential underestimation of the most important sADRs. We have focused on the analysis of sADRs through the investigation of PTs in BW section in the subsequent analysis.

### Drug induced organ toxicity

**Fig. 2:**
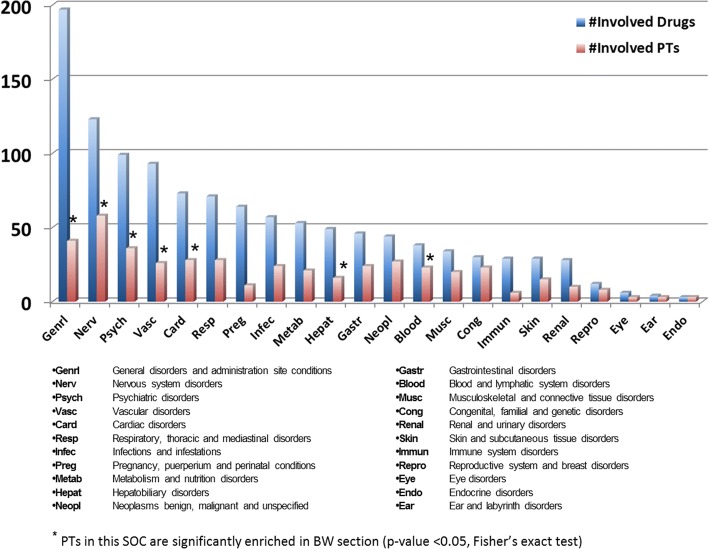
Number of PTs and Drugs involved in each SOC. SOCs were sorted by their involved drugs. Bars represent the number of Drugs and PTs involved in each SOC, respectively. Blue starred SOCs represent the PTs in these SOCs that are significantly enriched in BW section (*p*-value < 0.05, Fisher’s exact test)

To investigate drug toxicity at an anatomical organ/system level, we mapped the 460 Boxed Warning PTs to 22 disorder MedDRA System Organ Classes (SOCs). The statistical significance of PTs in a specific SOC was calculated using Fisher’s exact test. The number of PTs present in each SOC was plotted along with the number of drugs associated with those PTs in each SOC (Fig. 2). Out of the 22 SOCs, 7 were found to have PT enriched BW sections compared to the other 15 SOCs (*p* < 0.05) (Additional file 2) These 7 SOCs are General disorders and administration site conditions (*Genrl*), Nervous system disorders (*Nerv*), Psychiatric disorders (*Psych)*, Vascular disorders (*Vasc)*, Cardiac disorders (*Card*), Hepatobiliary disorders (*Hepat)*, and Blood and lymphatic system disorders (*Blood*). Furthermore, *Nerv* and *Vasc* BW sections were also statistically significantly enriched (*p*-value < 0.001). For example, drugs with *Hepat* enriched PTs are highly associated with severe drug induced liver injury (DILI). Among 50 drugs, 29 drugs are in the Liver Toxicity Knowledge Base (LTKB) [34], with 24 are considered among the most concerning DILI drugs [35].

Of note, SOC *Genrl* involved the highest number of drugs (197) and had 41 unique PTs like *Death*, *Pain*, and *Perforation*. SOC *Nerv* involved the second highest number of drugs (123) and contained the most PTs (58 unique PTs). SOCs *Card*, *Vasc*, and *Blood* involved a relatively higher number of drugs and a significantly higher number of PTs compared to SOCs Endocrine disorders (*Endo*), Eye disorders (*Eye*), and Ear and labyrinth disorders (*Ear*).

### Hierarchical cluster analysis reveal PT patterns across drug classes

**Fig. 3:**
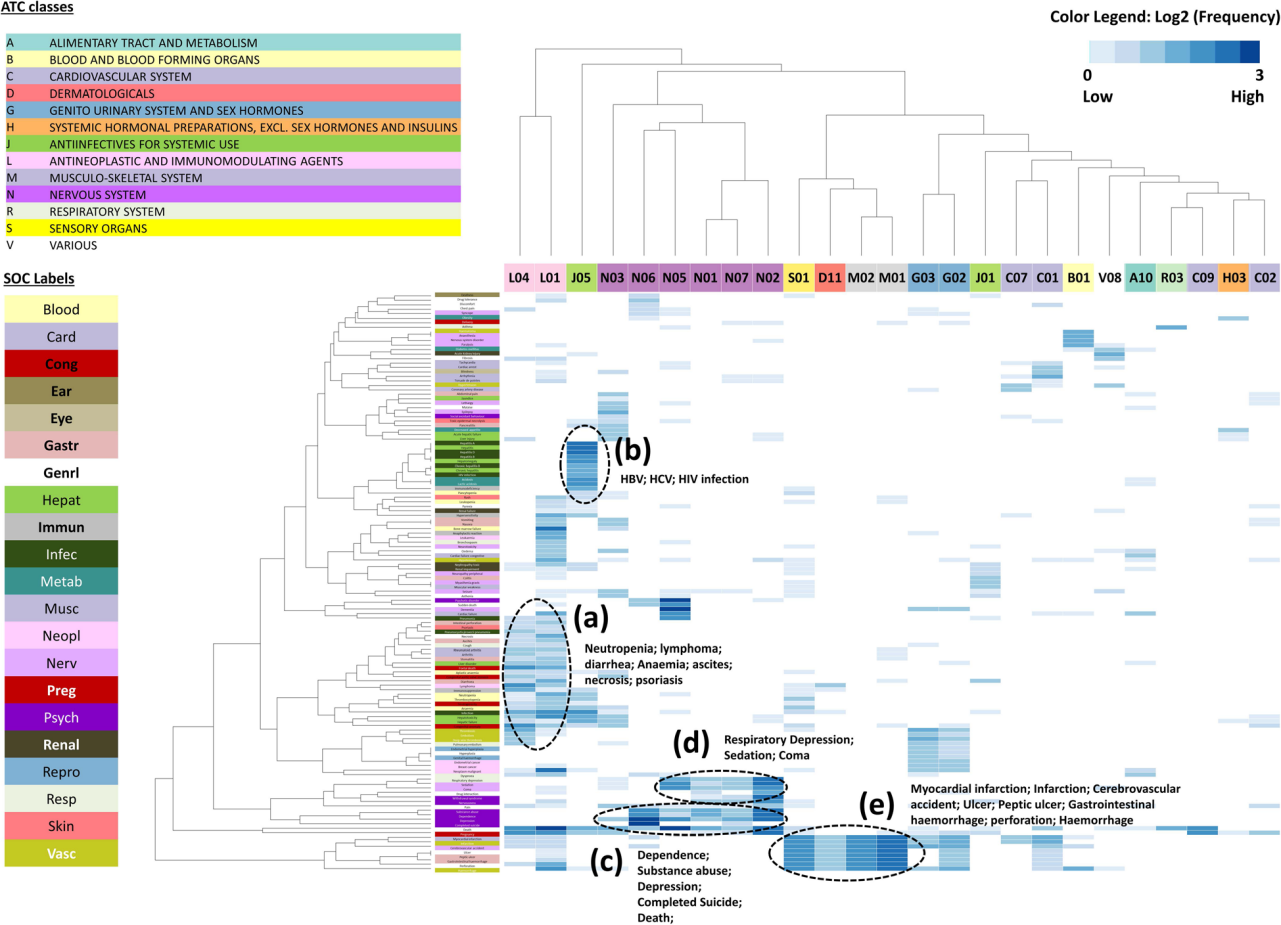
Clustering analysis results of drug ATC/MedDRA PT association. Only sADRs in BW section were analyzed. X-axis corresponds to drug ATC groups and Y-axis corresponds to MedDRA PTs. We analyzed MedDRA PTs and ATCs that were found to be associated with at least 5 drugs shown in the analysis. Drug classes and SOCs are represented by different colors. For example, PTs from SOC *Hepat* are green and can be identified in the Y-axis, whereas drugs from nervous system (N) are purple and can be identified at the X-axis

We further examined PT patterns in the Boxed Warning sections (BW) across different therapeutic classes identified using Anatomical Therapeutic Chemical (ATC) codes. Hierarchical Cluster Analysis (HCA) was performed with 129 PTs and 25 ATC groups. As shown in Fig. 3, two ATC classes, L01 (antineoplastic agents) and L04 (immunomodulating agents) were notably different from the other ATC groups with respect to the diversity of PTs belonging to L class (antineoplastic and immunomodulating agents). L01 (antineoplastic agents), the largest ATC group in our drug list, contained 50 drugs; whereas L04 (immunomodulating agents) contained 15 drugs. L01 (antineoplastic agents) involved 75 of the total 129 PTs including *neutropenia*, *lymphoma*, *diarrhea*, *anemia*, *ascites*, and *necrosis*. Both shared diverse PT profiling with 39 PTs (Fig. 3, cluster a). The wide coverage of PTs in L class (antineoplastic and immunomodulating agents) is consistent with the common knowledge that cancer drugs are associated with diverse adverse events [36].

### The same drug classes shared similar PT patterns

By applying HCA, we were able to investigate whether drugs under the same ATC therapeutic categories share similar PT patterns. HCA results revealed several clusters: **(a)** L01 (antineoplastic agents) and L04 (immunomodulating agents) shared diverse PT profiling with 39 PTs; L01 involved 75 of the total 129 PTs. **(b)** J05 (antivirals for systemic use) drugs were highly enriched with PTs like *Hepatitis* and *HIV infection*. **(c)** Nervous system ATC groups (N) were enriched with drug abuse related PTs like *substance abuse*, *dependence*, and *completed suicide*. **(d)** PTs such as *coma*, *respiratory depression*, and *sedation co*-*occurred* in BW of Nervous system drugs. **(e)** PTs such as *myocardial infarction* and *ulcer* were shared between S01 (ophthalmologicals), D01 (other dermatological preparations), M01 (anti-inflammatory and antirheumatic products), and M02 (topical products for joint and muscular pain), which all include NSAIDs that can increase the risk of serious gastrointestinal adverse reactions.

**Fig. 4:**
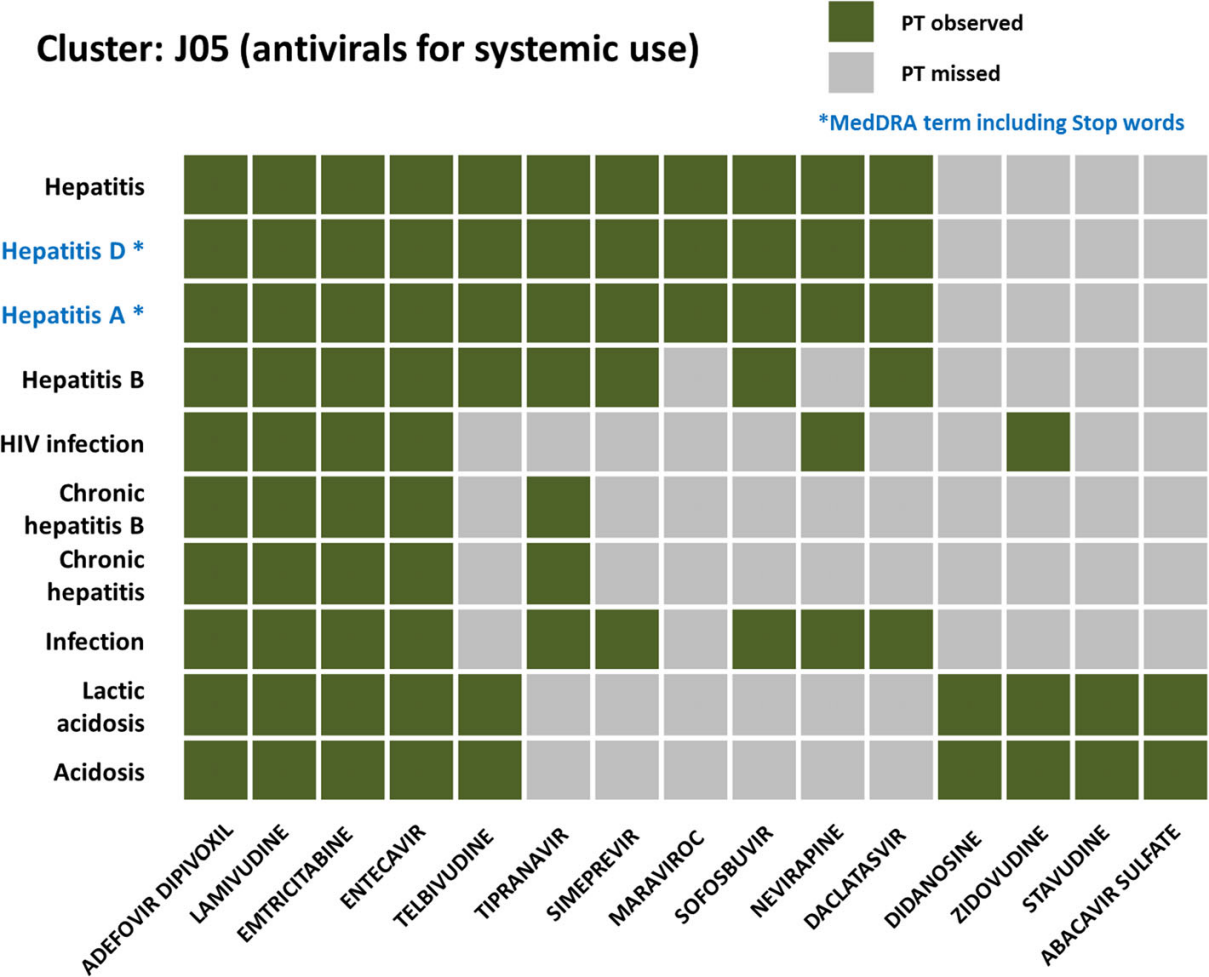
PT occurrence in cluster J05 (antivirals for systemic use) drugs. Only PTs in Fig. 3(b) cluster were further analyzed and were found to belong to two SOCs (Hepatobiliary disorders and Infections and infestations). It is noted that PTs with ‘*’ (Hepatitis D and Hepatitis A) include stop words (A and D), which are removed in Oracle Text queries, so they were queried and resulted the same as “Hepatitis”

For example, we found that in cluster b, J05 (antivirals for systemic use) was highly associated with PTs like *Hepatitis*, *Hepatitis A*, *Hepatitis B*, *HIV infection, Acidosis* and *Lactic acidosis* (Fig. 3, cluster b), all of which were observed in four J05 drugs (Adefovir dipivoxil, Lamivudine, Emtricitabine, and Entecavir). The remaining J05 drugs were categorized into two sub-groups where one group associated with hepatitis related PTs, and the other one associated with acidosis related PTs (Fig. 4). Regarding to the cluster c, all ATC classes in Nervous system drug (N) were enriched with drug abuse related PTs like *substance abuse*, *dependence*, and *completed suicide* (Fig. 3, cluster c)*.* Moreover, PTs such as *coma*, *respiratory depression*, and *sedation* were highly co-occurred in Nervous system drugs (Fig. 3, cluster d). Furthermore, we also observed an organ correlation of PTs in SOCs and drug classes. For example, the Nervous system class of drugs shared two sets of PTs **(**Fig. 3, cluster c and cluster d**)** belonging to Psychiatric disorder and Nervous system disorder, respectively.

## Discussion

Analysis was conducted on ADRs which were extracted from BW, WP, and AR sections using MedDRA terminology and Oracle Text search. We first conducted a comparative analysis of three ADR sections of drug labeling (i.e., BW, WP and AR). Next, we applied pattern recognition and statistical methods to analyze sADRs from BW across MedDRA SOCs and therapeutic classes to gain an understanding of the sADRs underpinning drug safety. Our study has shown that MedDRA hierarchical structure facilitates the novel use of drug labeling documents for the analysis of sADRs. In addition, data mining by combining MedDRA and drug class information revealed patterns of sADRs within and across ATC drug classes.

The number of MedDRA PTs occurring in each section increased in the order of BW < WP < AR while the severity of the ADRs decrease in the same order (BW > WP > AR). We compared the top 20 most frequently occurring MedDRA PTs among BW, WP, and AR. The six PTs (*Death*, *Pregnancy*, *Depression*, *Hemorrhage*, *Cardiac Failure*, *Infection*) that overlapped between BW and WP are more serious ADRs in comparison to eight PTs (*Nausea*, *Pain*, *Vomiting*, *Diarrhea*, *Hypersensitivity*, *Pyrexia*, *Infection*, and *Hypertension*) that were highly present in both WP and AR. We noticed that only one PT (*Infection*) out of 20 top PTs was present across all three sections, indicating that virus infection could lead to diverse side effects of drug use.

Analysis results showed that a PT occurring in different sections may carry a different frequency and weight. For example, PT *Myocardial infarction* occurred 34/367 (9.26%) times in BW sections and was observed 193/1148 (16.8%) times in WP sections, indicating that the usage frequency of *Myocardial infarction* is similar in the two labeling sections, mainly because sADRs like *myocardial infarction* are described in both BW and WP. On the other hand, PT *Hypersensitivity* showed a different rate among the sections, as it only occurred 11/367 (3.00%) times in BW sections but occurred 360/1148 (31.36%) times in WP sections. *Hypersensitivity*’s appearing more often in WP than BW section indicates that the seriousness of *Hypersensitivity* varies from drug to drug. Thus, the frequency and seriousness of the ADR will need to be taken into consideration while evaluating ADR risks.

Most, if not all, previous ADR studies using drug labeling with MedDRA [30, 32] focused on ADRs from the entire drug labeling with no discrimination in the severity of the same ADRs appearing in different sections. Such an approach does not fully take advantage of the drug labeling information. For example, SIDER is a well-established resource containing information on marketed medicines and recorded ADRs, which is mainly extracted from public documents and drug labeling. The available information includes ADR frequency, drug and ADR classifications, drug indication, and other relevant information. However, the SIDER database does not discriminate ADRs of one section from another, which could lead to a false representation of ADRs. The separation of ADRs by sections is of great importance when discriminating the seriousness level of ADRs for drug safety monitoring and evaluation [14, 35], as shown in this study.

HCA analysis revealed that the same classes of the drugs are likely to have similar PT (i.e., ADR) patterns. Drugs from sub-therapeutic categories N01 and N02 (e.g., opioids) in the Nervous system class (N), were more related to PTs such as *substance abuse*, *dependence* (including LLT *addiction*), and *respiratory depression* (Additional file 3). These findings are consistent with our understanding that the opioid crisis is highly related to addiction. The opioid epidemic is one of the most pressing public health concerns in the U.S. and is a top priority for the FDA [37]. For drugs that are known to have potentially serious risks, the FDA has enhanced labeling by incorporating the Risk Evaluation and Mitigation Strategy program (REMS) to provide an oversight for the continued safe use of those drugs [38]. One N01 drug (fentanyl) and two N02 drugs (buprenorphine, oxycodone) are opioids under REMS (Fig. 3, cluster c). Another N01 drug involved in cluster c (sodium oxybate) is also under REMS.

In this study, we applied MedDRA terms to extract ADRs in drug labeling, an area that has not been well investigated. Drug labeling documents are in free text, making it difficult to extract information and conduct ADR analysis. Use of MedDRA terminology to standardize ADR terms helps to enhance the analytical ability in text mining. This method can be deployed in pharmacovigilance by mining free text observational data for adverse drug events to assist drug safety surveillance. In addition to MedDRA, there are other biomedical terminologies, dictionaries, and coding systems (e.g., SNOMED-CT and ICD9) that have been developed for public healthcare information dissemination [39]. However, SNOMED-CT is not limited to tractable levels for its hierarchies (i.e., more than 10 levels), which creates hurdles for the translational and regulatory application. The MedDRA hierarchy, with five clearly defined levels, simplifies mapping and coding practices and facilitates communications with ADR reporting systems like the FDA Adverse Event Reporting System (FAERS). Of note, MedDRA is used as the adverse event reporting terminology by many drug regulatory authorities and the pharmaceutical industry worldwide but is not required for FDA-approved drug labeling. MedDRA PTs can be used to describe medical events and medication errors that are AEs or ADRs.

To evaluate Oracle Text search performance on MedDRA terms extracted from the Boxed Warning drugs, we compared our results with a dataset of manually extracted ADRs from 200 drug labeling published in Scientific Data in 2018 (as a gold-standard dataset) [40]. Specifically, our study and the publication had 30 BW drugs in common. We calculated the recall and precision for each drug (Additional file 4). On average per drug, the recall score for PTs was 0.93 by Oracle Text search; 26 of the 30 (86.7%) drugs yielded 1.0 recall. Four of the 30 drugs had false-negative PTs (total of 3 different PTs). Differences were due to identification of PTs which occurred during the manual coding by experts (using human interpretation) in the reference dataset, that Oracle Text search was unable to match because those words did not appear in that exact order in the labeling text (details see Additional file 4). For example, Oracle did not recognize the term “*suicidal behavior”* when it occurred in the text as “*suicidal* thinking and *behavior*.” The average precision was low, 0.46, indicative of high false-positives, which were mostly contributed to the occurrence of an extra smaller term within a larger term (e.g., *myocardial infarction* contains PT term *infarction*) which is difficult for Oracle Text to distinguish as one larger PT and not two PTs.

Further caution should be exercised due to the following listed reasons. First, drug labeling documents are not mandated to be MedDRA coded and some ADRs in drug labeling are worded differently from the terms in MedDRA which could cause Oracle Text query to fail to identify them. Second, MedDRA has terms beyond ADRs for regulatory reporting purposes. Third, stop words and multiple-meaning words may pose an additional limitation. Oracle Text query was built with basic NLP (Natural Language Processing) techniques including stop word removing, stemming, and tokenization. Default stop words used during Oracle Text indexing and mapping of the MedDRA dictionary did present a problem. For example, *Hepatitis A* contained the stop word ‘A’ and *Hepatitis D* contained the stop word ‘D’. Thus, all labeling that contained “Hepatitis *” was identified as a positive hit regardless of whether it was A, D, or another stop word (Fig. 4). Lastly, issues with multiple-meaning words were also identified during this study. For example, drug labeling might contain the word “fall” as in “fall in hemoglobin,” meaning decreased blood hemoglobin level. Therefore, the accurate coding for this situation should be LLT “*Hemoglobin decreased*” not LLT “*fall*,” which refers to a person “falling down.”

Overall, relatively high recall and low precision was observed using Oracle Text search compared to the gold standard MedDRA manually coded, which indicates that automatic computer programs could help identify and narrow ADR terms to reduce labor-intensive manual coding. However, manual validation is essential to reduce false-negatives and false-positives. In addition, further refinement of Oracle Text (e.g., advanced NLP) search based on the understanding of the MedDRA standard and Drug labeling text documents is warranted.

## Conclusion

This study demonstrated that combining MedDRA standard terminologies with data mining techniques facilitated computer-aided ADR analysis of drug labeling. This study also highlighted the importance of discrimination of the same ADRs which appear in different labeling sections. We specifically focused on serious ADRs primarily presented in BW as a proof-of-concept for the study of ADRs and the same approach should be equally applicable to other public health documents. It is worthwhile to point out that the proposed approach can be developed with consideration of other labeling sections, such as Indications and Usage, Drug Interactions, Contraindications, and Clinical Studies, to extract valuable safety and efficacy related information from drug labeling documents and even other public health documents (e.g., Electronic Health Records).

## Materials and methods

### Drug labeling documents

Drug labeling documents used in this study are in the Structured Product Labeling (SPL) format. SPL is a document markup standard approved by the Health Level Seven International (HL7), mandated by the FDA since 2005, as a standard XML format used to guide manufacturers on how to report and share drug product information. A wealth of material associated with a drug is included in the SPL (e.g., text, tables, safety and use information, active ingredients, package inserts, packaging type), and is required for all human drug products, including over-the-counter and biologic drug products. The FDA’s Center for Drug Evaluation and Research manages SPL submissions and approvals for US marketed drug products. In SPL documents, each labeling section title is coded by Logical Observation Identifiers Names and Codes (LOINC), which is a set of universal codes used to identify or exchange medical information. For example, the LOINC code for BW is 34,066–1, and the LOINC code for WP is 43,685–7. We used LOINC to parse the three ADR related sections (BW, WP, AR) from the XML-based SPL file.

### FDALabel database

FDALabel database (https://www.fda.gov/scienceresearch/bioinformaticstools/ucm289739.htm) was used to collect the drug labeling documents for this study [13]. FDALabel is developed and maintained by the FDA as a web-based application that allows access to the most up-to-date drug-labeling data, aiding their use in regulatory science, drug development, and scientific research. In its latest version, FDALabel allows the easy querying of drug information based on labeling sections (e.g., BW, WP, and AR). SPL documents are the source of FDALabel and are archived by the FDA and can be downloaded from DailyMed [41]. The current version of FDALabel database (3/20/2017) has 94,657 SPLs, which include human prescription drugs, biological products, and over-the-counter (OTC) drugs.

### FDA-approved NDA drug list

In the current version of FDALabel, 34,681 of the 94,657 SPLs are of human prescription drug labeling (hereafter called “drug labeling”). Of note, one prescription drug can have multiple SPLs due to the differences in regulatory applications, dosage forms, routes of administration, manufacturers, etc. For this study, duplicates of SPLs with the same Unique Ingredient Identifier (UNII) were removed and only the most recent effective SPL of the UNII drug was used. The drug list used in this study was selected using the following sequential criteria: (I) human prescription drug; (II) New Drug Application (NDA) drug; (III) single active ingredient UNII; (IV) most recent SPL of the same UNII of a drug. Finally, 1164 unique drug SPLs were extracted. The detailed drug list is provided in Additional file 5.

### Extracting MedDRA standardized terms for ADR study using Oracle text search

In this study, version 19.0 was used and has, in total, 75,818 LLTs, 21,920 PTs, 1732 HLTs, 335 HLGTs, and 27 SOCs. MedDRA has anatomical, physiological, and etiological SOCs. AEs or ADRs coded by MedDRA LLTs are classified per MedDRA’s predefined hierarchy and can be aggregated using SOCs. Of the 27 SOCs, 22 are “disorder” SOCs with PTs that are highly related to ADRs, such as *Cardiac disorders* and *Psychiatric disorders*. We removed 5 SOCs that were not ADR specific: *Injury, poisoning and procedural complications* (*Inj&P*), *Investigations* (*Inv*), *Social circumstances* (*SocCi*), *Surgical and medical procedures* (*Surg*), and *Product issues* (*Prod*).

We extracted ADRs in drug labeling with LLTs through an Oracle Text querying strategy and then linked the LLTs to their corresponding PTs for frequency counting. We counted each PT only once per section per labeling, regardless of how many times the PT, or its subordinate LLTs, occurred within the specific labeling section. Although PTs can be linked to multiple SOCs, for our SOC level analysis, only the primary SOC was considered.

The MedDRA terms extraction process was conducted using Oracle Text search. First, the labeling SPLs of full text sections, as XML, were parsed into the Oracle database based on LOINC [13]. The text index was built in basic NLP procedures at Oracle database including stop word removal, stemming, and pattern matching [42, 43]. Then, the processed text information was indexed and extracted using MedDRA LLTs and mapped to PTs. Specifically, the LLTs and PTs were extracted for each drug labeling document from three ADR related sections (i.e., BW, WP, and AR) as well as the whole document using structured query language (SQL). The resulting drugs - PTs matrix was used for further data analysis.

### Fisher’s exact test of SOC significance

Fisher’s exact test was performed per individual SOC, comparing the number of PTs that occurred in BW drugs belonging to the SOC to the total number of PTs occurring in that SOC for the FDA-approved NDA drug list. Since multiple SOCs were tested, Bonferroni correction (*p* < 0.002) was further considered in determining whether SOCs had significantly enriched Boxed Warnings (Additional file 2).

### Anatomical therapeutic chemical (ATC) codes

Anatomical Therapeutic Chemical (ATC) classification system classifies drugs by organ or system of involvement, as well as by chemical, therapeutic, and pharmacological properties. In this study, drugs were categorized into 54 ATC classes under therapeutic/pharmacological levels (the second level in ATC hierarchy). Details can be found in Additional file 6. If a drug had multiple ATC codes, all ATCs were counted separately. ATC information for the 1164 drugs was retrieved from the DrugBank database [44]. First, we mapped via the active ingredient, then we mapped the remaining drugs to Active moiety UNIIs. Thus, 989 drug-ATC relationships were identified and used to group the drugs into ATC classes.

### Hierarchical clustering analysis

A two-way Hierarchical Cluster Analysis (HCA) is an unsupervised learning approach and primarily used for pattern discovery [45]. In this analysis, HCA was used to investigate the grouping of ADRs (along with associated PTs) for BW drugs (i.e., drugs with a BW) in terms of their similarities across drug classes (ATC). Log 2 transformations of PT frequencies were performed to conduct the HCA analysis. Extracted PT data and ATC group data were organized into a data matrix where each row represented a single MedDRA PT, and each column represented an ATC secondary-level group. The frequency of each PT is the number of drugs in one ATC group that contained this PT in the labeling.

Some ATC groups have multiple drugs, such as antineoplastic agents (L01), psycholeptics (N05), and psychoanaleptics (N06). However, some ATC groups only contain one BW drug, such as antifungals for dermatological use (D01) and pituitary and hypothalamic hormones and analogues (H01). To reduce possible data noise in low frequency values, we compiled a preprocessed data matrix containing only ATC groups with at least 5 drugs, which were then further explored by cluster analysis. Similarly, only PTs that appeared in at least 5 drug counts across all drugs were included in the cluster analysis. Overall, for the final analysis, 129 out of 460 PTs and 25 out of 54 ATCs were used to compile a preprocessed data matrix (Additional file 7), and were analyzed by cluster analysis using *heatmap.1* function in R (version 3.2.1).

## Additional files

Additional file 1: **Table S1.** The entire MedDRA PT corpus for Boxed Warning sections among selected 367 drugs; (XLS 62 kb)

Additional file 2: **Table S2.** Drugs and PT distributions among MedDRA SOCs (XLS 33 kb)

Additional file 3: **Table S3.** Enriched cluster among ATC N categories (XLS 40 kb)

Additional file 4: **Table S4.** MedDRA term extraction performance of Oracle Text Search (XLS 56 kb)

Additional file 5: **Table S5.** 1164 selected SPL documents used in this study. (XLS 378 kb)

Additional file 6: **Table S6.** Overview of ATC second-level involved drugs (XLS 42 kb)

Additional file 7: **Table S7**. Detailed PT distributions among drug ATC categories (XLS 66 kb)

## Abbreviations

ADR: Adverse drug reaction
AR: Adverse reactions
BW: Boxed warning
HLGT: High level group term
HLT: High level term
LLT: Low level term
MedDRA: Medical dictionary for regulatory activities
NDA: New drug application
NLP: Natural language processing
PT: Preferred term
sADR: Serious adverse drug reaction
SOC: System organ class
SPL: Structured product labeling
SQL: Structured query language
UNII: Unique ingredient identifier
WP: Warnings and precautions
